# Supplementary material for: Information loss and bias in likert survey responses
Source: PLoS One. 2022 Jul 28;17(7):e0271949. doi: 10.1371/journal.pone.0271949 (PMC9333316; doi:10.1371/journal.pone.0271949)
Supplement: Attachment — Submitted filename: Rebuttal-Letter-for-PONE-D-21-01685.pdf [file pone.0271949.s001.pdf]

Rebuttal Letter for PONE-D-21-01685

J. Christopher Westland

6/15/2021

To the editors of PLOS One:

A 'Rebuttal Letter' is a requirement by PLOS One for any manuscript resubmitted after a previous rejection, and should outline the response to the original reviewers' comments, so that the editors can establish whether the original concerns have been addressed. It should provide a point-by-point response to the comments provided by the original reviewers, outlining the revisions carried out since the original submission.

My previous version's rejection letter was received without reviewer comments, and appears to be the decision solely of the handling editor, Dr. Jeff Galak, PhD., as described in his letter to me (reproduced here).

```
PONE-D-21-01685 Information Loss and Bias in Likert Survey Responses PLOS ONE

Dear Dr. Westland,

Thank you for submitting your manuscript to PLOS ONE. After careful consideration, we have
decided that your manuscript does not meet our criteria for publication and must therefore be
rejected.

Specifically, though the paper topic is quite interesting and relevant, I am afraid it does not
meet the publication requirements for PLOS ONE. In particular, per the criteria for publication
(found here:  https://journals.plos.org/plosone/s/criteria-for-publication) this paper reads as
an opinion piece or commentary and lacks empirical support.  The modeling is interesting, but
entirely speculative and not grounded in new evidence.  If you wish to empirically validate your
model and re-submit the paper as a new submission, we would certainly welcome that.

I am sorry that we cannot be more positive on this occasion, but hope that you appreciate the
reasons for this decision.

Good luck with your research.

Yours sincerely,

Jeff Galak, PhD Academic Editor PLOS ONE
```

The revisions I have made to the current paper are substantial, as I have documented in my *Track Changes* version of my manuscript uploaded to my File Inventory as an 'Other' file.

The specific reason for rejection were PLOS One publication requirements at https://journals.plos.org/plosone/s/criteria-for-publication which suggests that this is an opinion piece. I am not sure I completely agree with this assessment, as my original submission presented a well-structured argument based on standard statistical and survey assumptions with examples to demonstrate potential biases that could be encountered in survey research. Nonetheless, I concur that my case would be made substantially more compelling by validating the normative arguments made in the modeling section with a large-scale empirical study of actual survey results. I have provided this large-scale 'natural experiment' in the current resubmission of the paper.

I will briefly delineate my response to the numbered paragraphs below.

1. Revised the introduction and literature review to be more concise and relevant to the research question.
2. Simplified and shortened the mathematical and axiomatic arguments for my model of Likert-scaled representations of actual beliefs.

3. Presented two equivalent definitions of information loss for the normative vs. empirical sections of the paper: (1) Kullback-Leibler divergence (KLD) between two distributions, where loss is the KLD of the emprical Likert-scaled vs. the idealized distribution of beliefs; and (2) Fisher Information which is the Hessian of KLD, for information in the parameters of a known distribution (used in the normative modeling).
4. Added survey section, with details of the survey, and summary statistics for the empirical dataset for all 14 questions.
5. Added empirical results section, with KLD density plotted for all 442 behavioral-demographic groups in the empirical dataset for all 14 questions.
6. Revised conclusions and discussions germane to current practice in Likert-scaled surveys, with suggestions for further improving control.

I hope you find that my additional material satisfies all of the requirements for resubmission. I look forward to contributing to PLOS One.

Best regards

Chris Westland
