# Supplementary material for: Information loss and bias in likert survey responses
Source: PLoS One. 2022 Jul 28;17(7):e0271949. doi: 10.1371/journal.pone.0271949 (PMC9333316; doi:10.1371/journal.pone.0271949)
Supplement: Attachment — Submitted filename: review.pdf [file pone.0271949.s002.pdf]

Review of
Information Loss and Bias in Likert Survey Responses. PONE-D-21-19317

September 1, 2021

1 Main points

The paper sets forth the task of examining distributional implications of Likert response surveys. The latter are widely used accross many social disciplines such as marketing, opinion polls, economic disciplines and health surveys among many others. Being discrete categorical metrics, they can not only lose significant information from the real-world mappings beliefs, but also generate significant biases into the statistical analysis. The manuscript sets forth the task of showing through probabilistic arguments when do Likert scales do what they are supposed to do and when things can go wrong (strong polarization and beliefs). Such problems practically disappear when beliefs are Normal beliefs in Likert scaled surveys. The Normal fit works pretty well under many circumstances. The manuscript recommends using Likert-scaled surveys to allow minimal bias and information loss

2 Recommendation

I think the topic is very important for many scientific studies that researchers know about how to work better using Normal Likert scaled surveys. I think it would be useful to include some recent litterature that have takcled to model customer preferences from a different perspective to avoid the shortcomings of loss of information and biases using Likert scales. An example of paper is one on an alternative Bayesian modelling of Likert distributions such as Reinoso-Carvalho et al. (2020). They use discrete scale that in practice in contious by the use of a Bayesian Logit-Normal distribution. In this sense, there has been an interesting discussion from the Bayesian perspective and litterature dealing with such problems posed by the Likert scales and it would be interesting that the author fills that discussion in the introduction and motivation of the manuscript to make it more complete. Based on what was said above I recommend a revise and resubmit.

References

Reinoso-Carvalho, F., Gunn, L., ter Horst, E. and Spence, C. (2020). Sonic seasoning in the absence of a comparison stimulus: Opening possibilities for greater applicability. *Foods*, **9** 1–20.
