# Supplementary material for: Information loss and bias in likert survey responses
Source: PLoS One. 2022 Jul 28;17(7):e0271949. doi: 10.1371/journal.pone.0271949 (PMC9333316; doi:10.1371/journal.pone.0271949)
Supplement: Attachment — Submitted filename: review.pdf [file pone.0271949.s004.pdf]

Review of Information Loss and Bias in Likert Survey Responses. PONE-D-21-19317.R1

June 16, 2022

1 Main points

The paper sets forth the task of examining distributional implications of Likert response surveys. The latter are widely used accross many social disciplines such as marketing, opinion polls, economic disciplines and health surveys among many others. Being discrete categorical metrics, they can not only lose significant information from the real-world mappings beliefs, but also generate significant biases into the statistical analysis. The manuscript sets forth the task of showing through probabilistic arguments when do Likert scales do what they are supposed to do and when things can go wrong (strong polarization and beliefs). Such problems practically disappear when beliefs are Normal beliefs in Likert scaled surveys. The Normal fit works pretty well under many circumstances. The manuscript recommends using Likert-scaled surveys to allow minimal bias and information loss

2 Recommendation

I have reviewed the manuscript and it has improved substantially from the first version. I am happy to recommend it for publication. The only minor detail is a reference regarding Reinoso-Carvalho et al. (2020). Please change the author name "Horst E ter" to "ter Horst E".

References

Reinoso-Carvalho, F., Gunn, L., ter Horst, E. and Spence, C. (2020). Sonic seasoning in the absence of a comparison stimulus: Opening possibilities for greater applicability. *Foods*, **9** 1–20.
